# Melatonin Derivative-Conjugated Formulations of Pd(II) and Pt(II) Thiazoline Complexes on Mesoporous Silica to Enhance Cytotoxicity and Apoptosis against HeLa Cells

**DOI:** 10.3390/pharmaceutics16010092

**Published:** 2024-01-10

**Authors:** Samuel Estirado, Diana Díaz-García, Elena Fernández-Delgado, Emilio Viñuelas-Zahínos, Santiago Gómez-Ruiz, Sanjiv Prashar, Ana B. Rodríguez, Francisco Luna-Giles, José A. Pariente, Javier Espino

**Affiliations:** 1 Grupo de Investigación Neuroinmunofisiología y Crononutrición, Departamento de Fisiología, Facultad de Ciencias, Universidad de Extremadura, Avenida de Elvas s/n, 06006 Badajoz, Spain; estirado@unex.es (S.E.); elenafd@unex.es (E.F.-D.); moratino@unex.es (A.B.R.); pariente@unex.es (J.A.P.); 2 COMET-NANO Group, Departamento de Biología y Geología, Física y Química Inorgánica, E.S.C.E.T., Universidad Rey Juan Carlos, Calle Tulipán s/n, Móstoles, 28933 Madrid, Spain; diana.diaz@urjc.es (D.D.-G.); sanjiv.prashar@urjc.es (S.P.); 3 Grupo de Investigación Química de Coordinación, Departamento de Química Orgánica e Inorgánica, Facultad de Ciencias, Universidad de Extremadura, Avenida de Elvas s/n, 06006 Badajoz, Spain; emilvin@unex.es (E.V.-Z.); pacoluna@unex.es (F.L.-G.)

**Keywords:** melatonin, palladium, platinum, silica, drug delivery, HeLa

## Abstract

The search for alternatives to cisplatin has led to the development of new metal complexes where thiazoline derivatives based on platinum(II) and palladium(II) stand out. In this sense, the Pt(II) and Pd(II) complexes coordinated with the thiazoline derivative ligand 2-(3,4-dichlorophenyl)imino-N-(2-thiazolin-2-yl)thiazolidine (TdTn), with formula [PtCl_2_(TdTn)] and [PdCl_2_(TdTn)], have previously shown good results against several cancer lines; however, in this work, we have managed to improve their activity by supporting them on mesoporous silica nanoparticles (MSN). The incorporation of metal compounds with a melatonin derivative (5-methoxytryptamine, 5MT), which is a well-known antioxidant and apoptosis inducer in different types of cancer, has been able to increase the cytotoxic activity of both MSN-supported and isolated complexes with only a very low amount (0.35% *w*/*w*) of this antioxidant. The covalently functionalized systems that have been synthesized are able to increase selectivity as well as accumulation in HeLa cells. The final materials containing the metal complexes and 5MT (MSN-5MT-PtTdTn and MSN-5MT-PdTdTn) required up to nine times less metal to achieve the same cytotoxic activity than their corresponding non-formulated counterparts did, thus reducing the potential side effects caused by the use of the free metal complexes.

## 1. Introduction

Since the discovery of cisplatin as an anticancer drug, much research has focused on the synthesis of platinum-based complexes as potential alternative metallodrugs against cancer [1]. Thiazoline derivate-containing Pt(II) and Pd(II) complexes (e.g., PtTdTn and PdTdTn) have already demonstrated strong pro-apoptotic capacity against different cancer lines such as HT-29 and U-937 [2]. However, the real biological application of these metal complexes still involves a series of drawbacks such as the need of the use of high doses due to their possible hydrolyzation, low stability in biological media and low selectivity capacity.

It is well known that, currently, drug delivery research is directly linked to the development of nanotechnology and, more specifically, to the search for systems based on nanoparticles with a high functionalization capacity [3]. Silica nanoparticles have several beneficial properties that make them perfect candidates for drug delivery such as suitable biodegradability, biocompatibility, mesoporous structure for high drug loading and chemical and mechanical stability [4,5]. The incorporation of metal complexes in this type of nanoparticulate systems allows us to solve most of the disadvantages associated with the use of free metal complexes, improving their bioavailability [6,7].

In this context, there are several ways of incorporating metal complexes in nanostructured silica-based materials to obtain drug delivery systems [8]. The most common is the preparation of “classical” systems, which consists of a simple adsorption of the complex in the pores of the silica [6]. This classical loading method improves the quantity of cargo in the nanomaterial; however, it requires “gates” to control the release of the drug not earlier than desired [9,10]. On the other hand, an alternative approach for the incorporation of the metal complexes into silica is the use of covalent binding with the silica nanostructure. These so-called “non-classical” systems do not require a release of the cytotoxic cargo but may act directly inside the cell due to accumulation in the affected area and internalization [11]. Using this covalent functionalization procedure, the incorporation of other molecules of interest, e.g., a targeting molecule, to increase the selectivity of the designed systems is possible [12,13,14]. For example, systems based on silica functionalized with proteins [15,16], vitamins [17,18,19] or sugars [20,21] have been prepared and have been shown to increase their biological activity mainly due to greater accumulation in the target area compared with that in those systems not having the targeting molecule.

Melatonin (N-acetyl-5-methoxytryptamine) is acknowledged as a powerful free radical scavenger and antioxidant. During the last few decades, the antitumor potential of melatonin has been examined [22]. Melatonin is considered an anticancer agent due to its anti-proliferative, anti-migratory and/or pro-apoptotic actions against various types of cancer, such as gastric [23,24], breast [25,26], cervical [27], and prostate cancer [28,29], among others. In addition, melatonin has been reported to provide enhanced antitumor effects in combination with chemotherapy drugs [26,30,31]. Nevertheless, melatonin is a poorly soluble hormone in aqueous solution, so its half-life, bioavailability, and distribution in cells are potentially limited in the biological environment [32]. This property may consequently pose a serious obstacle to achieving the desired therapeutic dose. Melatonin can be classified as a class II category drug (poorly soluble and highly permeable), according to the Biopharmaceutics Classification System [33,34]. Therefore, the use of melatonin-loaded nanostructures could be a good therapeutic strategy. In fact, in an attempt to achieve molecular protection and a sustained release of indoleamine, different types of nanoparticles have been used so far, e.g., solid lipids, biopolymers or polymeric vesicles, silica nanoparticles, nanofibers, or graphene nanocarriers, among others [35]. In terms of toxicity, silica is generally recognized as safe by the Food and Drug Administration (FDA) when used in in vitro or in vivo studies [36,37]. However, there is scarce literature on the loading of melatonin onto silica nanoparticles as drug carriers and most of the research in this regard is for non-pharmaceutical purposes [38,39], except for a few studies reporting remarkable in vitro release [40,41].

Along with melatonin, there is another type of pineal indole, 5-methoxytryptamine (5MT), which has been suggested to enhance cytotoxic activity against cancer [42,43,44,45]. It is, therefore, worth noting that 5MT may display anti-cancer properties, having, like melatonin, a variety of functions, including scavenging free radicals and inducing protective/reparative mechanisms in cells [46]. In this sense, the present work focuses on the covalent vehiculation of two metal complexes (PtTdTn and PdTdTn) in nanostructured systems based on selective mesoporous silica via the incorporation of the melatonin derivative 5MT. In addition, a thorough study of the cytotoxic capacity of these novel systems against human epithelial cervix carcinoma HeLa cells has also been carried out giving very promising results that validate the use of these materials as therapeutic agents.

## 2. Materials and Methods

### 2.1. General Remarks on the Synthesis and Characterization of the Materials

The reagents used in the preparation of the materials were tetraethyl orthosilicate (TEOS) and hexadecyltrimethylammonium bromide (CTAB), which were purchased from Aldrich. The ligands 3-mercaptopropyltriethoxysilane (MP) and N-(3-trimethoxysilylpropyl)ethylenediamine triacetic acid trisodium salt (TEDTS) were purchased from Fluorochem. The reagents for EDAC coupling, namely 1-ethyl-3-(3-dimethylaminopropyl)carbodiimide hydrochloride (EDAC) and N-hydroxysuccinimide (NHS), were purchased from Aldrich, while 5-methoxytryptamine (5MT) was purchased from Carbosynth. All the reagents were used without further treatment.

The compounds 2-(3,4-dichlorophenyl)imino-N-(2-thiazolin-2-yl)thiazolidine (TdTn), dichloro-[N (3,4-dichlorophenyl)-3-(4,5-dihydro-1,3-thiazol-2-yl)-1,3-thiazolidin-2-imine]-palladium(II) (PdTdTn) and dichloro-[N-(3,4-dichlorophenyl)-3-(4,5-dihydro-1,3-thiazol-2-yl)-1,3-thiazolidin-2-imine]-platinum(II) (PtTdTn) were prepared in accordance with known procedures [2,47].

For powder X-ray diffraction analysis, a Philips PW3040/00 X’Pert MPD/MRD spectrometer (Philips, Amsterdam, The Netherlands) was used, using mono-chromatic radiation. DR UV-vis measurements were collected with a Varian Cary-500 spectrophotometer equipped with an integrating sphere and polytetrafluoroethylene (PTFE) as a reference. Fourier-transform infrared spectra (FT-IR) were prepared using KBr pellets with a PerkinElmer FTIR Spectrometer. N_2_ gas adsorption-desorption isotherms (BET) were performed using a Micromeritics ASAP 2020 analyzer with a degassing process at 90 °C for 10 h. Thermogravimetry (TG) analyses were obtained with a DSC/TGA Discovery SDT650 under a nitrogen atmosphere. ICP-AES studies were carried out on Varian Vista AX Pro Varian 720-ES (λ (Pt) = 203.646 nm; λ (Pd) = 340.458 nm). Transmission electron microscopy (TEM) was carried out using JEOL JEM 1010 operating at 100 kV, and the micrographs were treated using ImageJ (version 1.53e). Scanning electron microscopy (SEM) was performed with XL30 ESEM Philips with high-resolution FEG-SEM Nova Nano SEM230 (SEM, North Billerica, MA, USA).

### 2.2. Synthesis of the Starting Mesoporous Silica Material (MSN)

The synthesis of the MSN was carried out, slightly modifying the experimental procedure reported by Zhao et al. [48]. The surfactant CTAB (2 g, 5.5 mmol) was dissolved in 480 mL of Milli-Q water (water purification system Milli-Q^®^ Direct, Merck, Darmstadt, Germany) at 1500 rpm. Subsequently, 7 mL of sodium hydroxide (2 M, 14 mmol) was added, and the temperature was adjusted to 80 °C; then, 10 mL of the silica precursor TEOS (44.8 mmol) was added dropwise, and the mixture stirred at 80 °C for 2 additional hours. The resulting white solid was separated via filtration, subsequently washed with abundant Milli-Q water and methanol (2 × 50 mL) and dried for 24 h at 80 °C in a stove. Finally, the surfactant was eliminated via calcination at 550 °C for 24 h.

### 2.3. Functionalization with Silane Ligands

**Figure 1:**
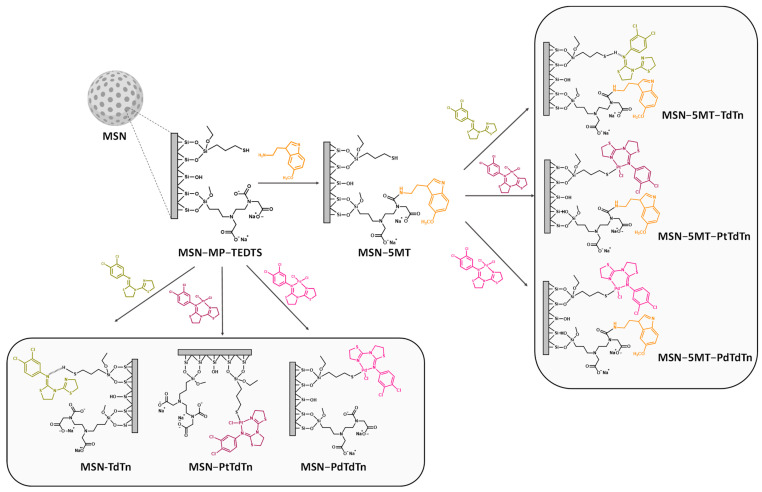
Schematic representation of the functionalization reactions for obtaining the six final materials.

The incorporation of the ligands MP and TEDTS was carried out in a two-step reaction (Figure 1). First, 1.5 g of MSN was dehydrated and activated under vacuum and at 80 °C overnight. The silica was dispersed in 40 mL of dry toluene, and 1.53 mL (6.33 mmol, 1:1 *w*/*w* SiO_2_:MP) of MP was added under a nitrogen atmosphere. The dispersion was kept under stirring at 110 °C for 48 h. After that time, the solid (MSN-MP) was isolated via centrifugation (6000 rpm, 10 min), washed several times with toluene and diethyl ether, and dried in a stove at 75 °C.

For the formation of MSN-MP-TEDTS, 1.3 g of MSN-MP was dried under vacuum and 80 °C overnight. The activated silica was dispersed in 30 mL of dry ethanol and 206.3 µL (0.56 mmol, 20% *w*/*w* SiO_2_:TEDTS) of TEDTS was added under a nitrogen atmosphere. The suspension was then heated to 60 °C and stirred for 48 h. The solid was then cooled to room temperature and the solid was then separated (6000 rpm, 10 min), washed with abundant Milli-Q water and methanol (2 × 50 mL), and dried for 24 h at 80 °C in a stove.

### 2.4. Incorporation of 5MT Derivative

For the functionalization of the materials with the melatonin derivative 5MT, an EDAC-mediated coupling reaction was carried out (Figure 1). Briefly, 80 mg of 5MT (0.42 mmol, 10% functionalization *w*/*w* SiO_2_/5MT) was dissolved in 2 mL of DMSO in an ultrasonic bath, and this solution was added to 80 mL of MES buffer. After obtaining a homogeneous mixture, 96 mg (0.50 mmol) of EDAC and 144 mg (1.25 mmol) of NHS were added and the resulting solution was stirred for 15 min at room temperature to activate the carboxylic groups. Subsequently, 800 mg of MSN-MP-TEDTS was added to the EDAC solution, and the resulting mixture was stirred for 2 additional hours at room temperature. Finally, the resulting solid was centrifuged (6000 rpm, 10 min), washed with Milli-Q water and ethanol (2 × 50 mL) in order to remove the excess reagents, and subsequently dried in a stove at 75 °C. The resulting product was labeled MSN-5MT.

### 2.5. Functionalization with the Cytotoxic Compounds

The synthesis of the MSN-based materials functionalized with the cytotoxic agents TdTn, PtTdTn or PdTdTn was carried maintaining the same proportion of the TdTn ligand in each final material. For the formation of MSN-PtTdTn, 200 mg of MSN-MP-TEDTS and 40.15 mg of PtTdTn (10% functionalization *w*/*w* SiO_2_/TdTn) were added to a Schlenk tube and subjected to two cycles of vacuum/nitrogen (10 min/1 min). The solids were dispersed in 20 mL of dry toluene and, subsequently, 18.6 µL of triethylamine was added. The reaction mixture was then heated to 110 °C and stirred for 24 h. Afterwards, the solid was centrifuged (10 min at 6000 rpm) and subsequently washed with ethanol. For the preparation of MSN-PdTdTn, 200 mg of MSN-MP-TEDTS and 34.22 mg of PdTdTn (10% functionalization *w*/*w* SiO_2_/TdTn) were used. MSN-TdTn was synthesized via adsorption to be used as a comparative material using 200 mg of MSN-MP-TEDTS and 22.22 mg of TdTn (10% functionalization *w*/*w* SiO_2_/TdTn).

For the preparation of MSN-5MT-TdTn, MSN-5MT-PtTdTn and MSN-5MT-PdTdTn, the quantities and synthetic procedure were the same except for starting from the functionalized material MSN-5MT (Figure 1).

### 2.6. Stability Study of the Final Materials: Release Analysis

A metal leaching study was carried out to determine the concentration of Pt(II) or Pd(II) soluble species in simulated body fluid with phosphate-buffered saline (PBS; pH 7.4). Thus, 5 mg of the final materials (MSN-PtTdTn, MSN-PdTdTn, MSN-5MT-PtTdTn and MSN-5MT-PdTdTn) was suspended in 5 mL of buffer and incubated at 37 °C under 30 rpm in a Roto-Therm incubator (Benchmark Scientific, Sayreville, NJ, USA) for 3, 24 and 48 h. After each studied time, the suspensions were filtered with a nylon filter (0.2 µm) and the solutions were analyzed via ICP-AES in duplicate to determine the quantity of Pt(II) or Pd(II) leached to the solution during the incubation.

### 2.7. DNA Interaction Study

Fish sperm-deoxyribonucleic acid (FS-DNA) was purchased from Aldrich (St. Louis, MO, USA). A stock solution of FS-DNA was prepared by dissolving an appropriate amount of FS-DNA in PBS buffer (pH = 7.4) and storing it at 4 °C in the dark. The concentration of the DNA stock solution (0.08 mg/mL) was determined from the UV absorption spectrum at 260 nm using the molar absorption coefficient ɛ_260_ = 6600 M^−1^·cm^−1^. UV absorption spectroscopy experiments were conducted by adding the same concentration of DNA 0.08 mol/L to suspensions of MSN-PdTdTn and MSN-PtTdTn (2.5 mg/mL in a mixture ethanol/Tris buffer) and incubating them at different time intervals: 1, 2, 4 and 24 h. After each incubation time, the suspensions were filtered with a nylon filter (0.2 µm), which was then followed immediately by spectroscopic measurements. All measurements were performed at room temperature with an Analytik Jena Specord 200 spectrophotometer (Analytik Jena, Jena, Germany) between 200 and 500 nm.

### 2.8. Cell Culture and Treatments

Human epithelial cervix carcinoma cells (HeLa) were cultured in Dulbecco’s modified Eagle’s medium (DMEM) supplemented with 10% fetal bovine serum, 2 mM of L-glutamine and 100 U/mL of penicillin/streptomycin at 37 °C in a humidified incubator with 5% CO_2_. For experiments, the cells were trypsinized, seeded in culture plates and treated with MSNs suspended in a solution of distilled H_2_O/DMSO (50:50, *v*/*v*) at concentrations ranging from 10 to 500 µg/mL for 24 h. Suspensions of MSNs were dispersed with a sonicator to minimize aggregation before addition into the culture medium. Cells were also challenged with 1–100 µM of the free compounds (PtTdTn, PdTdTn and TdTn) and 0.5–5 mM of 5MT for 24 h. A control with DMSO (vehicle) was used.

### 2.9. In Vitro Cytotoxicity Assay

The cytotoxic effect of the free compounds and MSNs were assayed by means of the CellTiter 96^®^ AQueous One Solution Cell Proliferation Assay, which is based on the reduction of a tetrazolium compound. Cells were seeded in 96-well plates at a density of 8 × 10^3^ cells/well. After treating cultures for 24 h, assays were performed by adding 10 µL of the CellTiter 96^®^ AQueous One Solution Reagent directly to culture wells, incubating cells for 1 h at 37 °C, and then recording absorbance at 490 nm on a microplate reader (Infinite M200, Tecan, Grödig, Austria).

### 2.10. Determination of Apoptosis

Phosphatidylserine externalization was examined using an annexin V-FITC/Hoechst 33258 assay to assess whether or not apoptosis was induced. In a nutshell, stimulated cells were collected (3 × 10^5^ cells/mL), washed with PBS once, and centrifuged at 300× *g* for 5 min. The supernatant was then discarded, and the pellet was resuspended in 200 µL of binding solution containing 5 µL of annexin V-FITC. After incubation for 10 min at room temperature, the cells were then resuspended in binding buffer that included 10 µL of Hoechst 33258. A MACSQuant X flow cytometer (Miltenyi Biotec, Bergisch Gladbach, Germany) was used to analyze the double-stained cells immediately.

### 2.11. Cellular Uptake and Accumulation of MSNs

The cellular uptake of MSNs was observed via TEM. The cells were seeded into 100 mm Petri dishes and treated with 10 µg/mL of palladium-containing MSNs for 24h. Then, cells were trypsinized, washed with PBS and centrifuged at 500× *g* for 5 min. Afterwards, the supernatants were removed, the cell pellets were fixed in 2.5% glutaraldehyde, postfixed in 1% osmium tetroxide, dehydrated in graded series of ethanol, and embedded in epoxy resin. Ultrathin sections were then sliced and stained with toluidine blue. The internalization of particles and cellular ultrastructure changes were imaged under TEM JEOL JEM 1010 (JEOL USA, Peabody, MA, USA).

To verify to which extent palladium(II) and platinum(II) accumulated inside HeLa cells, a density of 5 × 10^6^ cells was seeded in 100 mm Petri dishes and treated with 100 µg/mL of MSN-PdTdTn, MSN-5MT-PdTdTn, MSN-PtTdTn or MSN-5MT-PtTdTn for 3 h. The cells were then trypsinized, washed with PBS, centrifuged at 500× *g* for 5 min and lysed with radioimmunoprecipitation assay buffer (RIPA). Finally, after digestion with 65% HNO_3_, cells were analyzed with ICP-MS 7900 (Agilent Technologies, Santa Clara, CA, USA).

### 2.12. Statistics

The mean and standard deviation (SD) of the data were computed. One-way analysis of variance (ANOVA) followed by Tukey’s test or Dunnett’s test (as indicated) was used to determine statistical significance to make comparisons among treatments. The dose–response curves of each compound were constructed to obtain the IC_50_ values by fitting the curve using a four-parameter, sigmoid dose–response equation. A statistically significant difference was defined as *p* < 0.05. GraphPad Prism 7.04 for Windows was the statistics software utilized.

## 3. Results and Discussion

### 3.1. Synthesis and Characterization of the Mesoporous Silica Materials

Three new series of nanomaterials functionalized with the cytotoxic agents TdTn, PdTdTn or PtTdTn were synthesized via the protonolysis of the MP ligand and a melatonin derivative (5MT), which was incorporated via an EDAC-mediated amidation reaction. All materials were subsequently thoroughly characterized through different physicochemical techniques.

**Figure 2:**
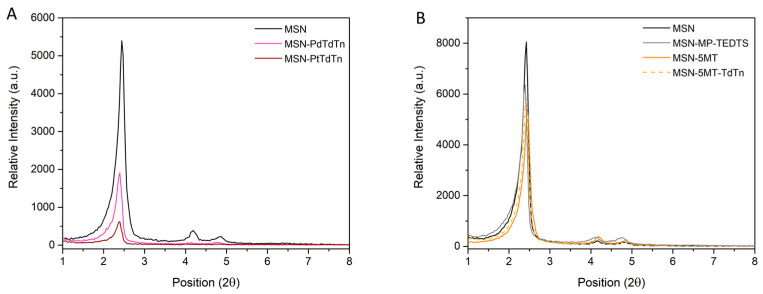
XRD patterns of some functionalized MSN materials with metallic complexes (**A**) and some 5MT series (**B**).

Figure 2 shows the XRD pattern of all prepared materials, which revealed an intense peak at ca. 2.4° corresponding with (100) Miller plane and two very low intensity peaks at 4.2° and 4.9°, corresponding to (110) and (200) planes, which is typical of an ordered mesoporous structure of the MCM-41 silica family. After the functionalization reactions with MP and TEDTS and, subsequently, with the 5MT ligand (Figure 2B), the intensities of the peaks clearly decreased due to the incorporation of the molecules inside the pores, which blocked the dispersion points of the materials, resulting in a clear detriment to the diffraction intensity. Indeed, one can clearly observe that the decrease in the relative intensity is more pronounced in the final materials, as can be seen in Figure 2A for the MSN-PdTdTn and MSN-PtTdTn systems. 

**Figure 3:**
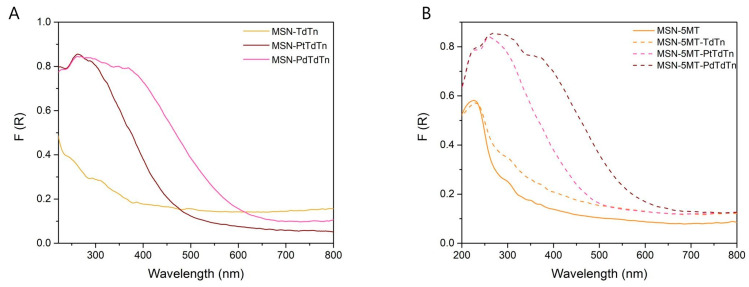
UV-vis spectra in solid state for MSN series with metal complexes or ligand without targeting molecule (**A**) and MSN series with metal complexes or ligand and with 5MT (**B**).

Solid-state DR UV-vis analysis supported the information obtained from the XRD measurements because after successive incorporations, the absorption bands were modified. Figure 3A shows how functionalization with a metal complex (PtTdTn or PdTdTn) produces an intense absorption band between ca. 250 and 400 nm for the ligand–metal charge transfer absorptions and for the d–d transitions, corresponding to the molecular d-orbital of the metal complexes [49]. The same behavior is found in the 5MT functionalized materials as shown in Figure 3B, where an additional absorption band around 228 nm, associated with the incorporation of the 5MT directing agent, is observed although it is obscured by the large absorption from the metal complexes.

The quantification of the functionalization of the different fragments has been carried out via thermogravimetry (TG) or via induction coupled plasma atomic emission spectroscopy (ICP-AES) (Table 1). The incorporation of the MP and TEDTS ligands determined via TG (7.12 mass % for MP and 5.27 mass % for TEDTS) shows functionalization rates similar to those of the other ligands incorporated under the same conditions on MSN [19]. Functionalization with the TEDTS ligand allowed the incorporation of the melatonin derivative 5MT via an EDAC-catalyzed amidation coupling. As shown in Table 1, the incorporated mass percentage of 5MT is relatively low (0.35%); however, the incorporation of this fragment to the nanostructured systems is sufficient to provide greater selectivity to the designed systems, as can be observed in the biological studies. In the same way, functionalization with MP allowed the incorporation of the cytotoxic agent (TdTn ligand) through adsorption via intermolecular interactions or the PdTdTn metal complexes, through a protonolysis reaction in the presence of NEt_3_.

**Figure 4:**
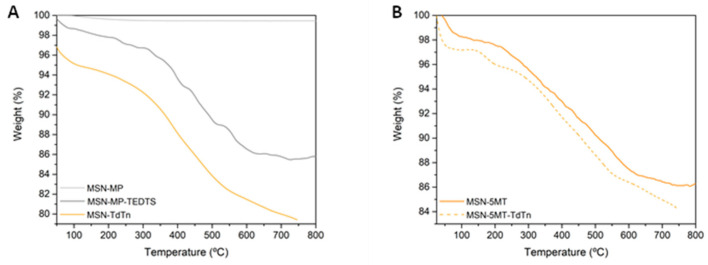
Thermogravimetric analysis of functionalized silica with ligands and TdTn (**A**) and 5MT with and without TdTn (**B**).

In addition, Table 1 shows the quantity of metal incorporated in the material, which is much lower when the system has 5MT (4 times lower than in the case of Pd(II) and almost 8 times lower than in the case of Pt(II)), since the surface area of these materials is smaller and the accessibility of the thiol groups to the metal complexes considerably decreases. The same behavior was observed with the incorporation of the TdTn ligand in the starting silica and in MSN-5MT where the calculation of the functionalization percentages for both the TdTn ligand and the 5MT target was carried out from the associated TG plots (Figure 4). In addition, thermal transformations observed in the DTG curves (Figure S1) gave an indication of the different processes associated with the sequential decomposition of the fragments attached to the silica. TG-DTG analysis of the materials with TdTn (Figure 4 and Figure S1A) shows up to 8-fold higher ligand incorporation when the silica is not functionalized with 5MT. However, comparing the quantity of TdTn in all materials (including the metal compounds), a higher percentage of TdTn is observed when it is part of a metal complex than when TdTn is the only agent incorporated in MSN (MSN-TdTn), which is probably due to the fact that the incorporation of metal complexes occurs via covalent bonding, increasing the stability and non-controlled release of the ligand (Table 1).

**Figure 5:**
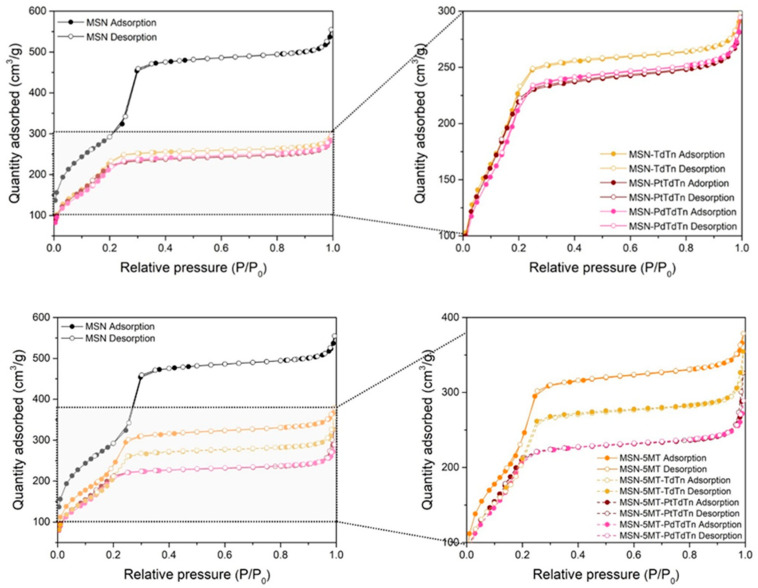
Adsorption–desorption isotherms of MSN and some functionalized materials.

To confirm the porous nature of the synthesized silicas and how the surface and porous features of the systems change after functionalization with the different agents, N_2_ adsorption-desorption isotherms were carried out (Figure 5). The isotherms were identified as type II according to the IUPAC classification [50]. MSN samples demonstrated an increase in the adsorbed nitrogen volume at P/P_0_ values around 0.2 and 1, along with the appearance of a small H1-type hysteresis loop [51], with a plateau at high P/P_0_, indicating nitrogen capillary condensation inside the mesopores. It is important to note that the studied MSN materials have a uniform inner structure with hexagonal pore distribution and a controlled diameter of about 2.8 nm (evaluated by Barrett-Joyner–Halenda (BJH)), which is not highly affected after several functionalization steps (Table 2). However, the pore volume and surface area of the silica (measured by Brunauer–Emmett-Teller (BET)) are affected after the functionalization. The surface area of the starting silica was higher than 1000 m^2^/g while, when incorporating the different fragments, it decreased by up to 1.4 times. A similar trend was found when analyzing the pore volume, which decreased to even more than half its capacity compared to that of the initial MSN (from 0.80 to 0.39 cm^3^/g in the case of the final material MSN-5MT-PdTdTn). These changes in surface parameters indicate that homogeneous functionalization took place both on the surface of the material and inside the pores.

**Figure 6:**
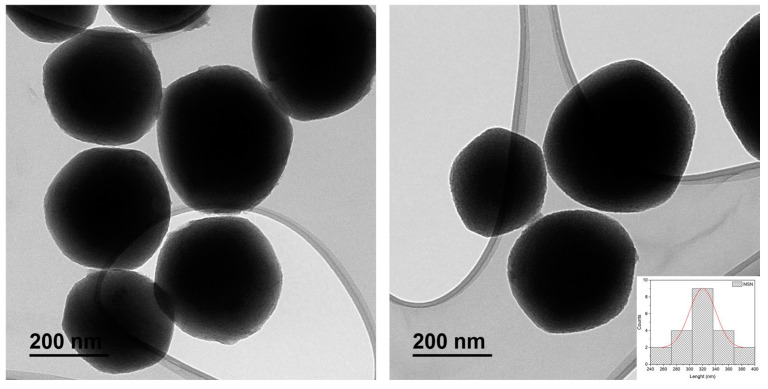
TEM images of starting MSN and histogram of particle size distribution with gaussian fit.

**Figure 7:**
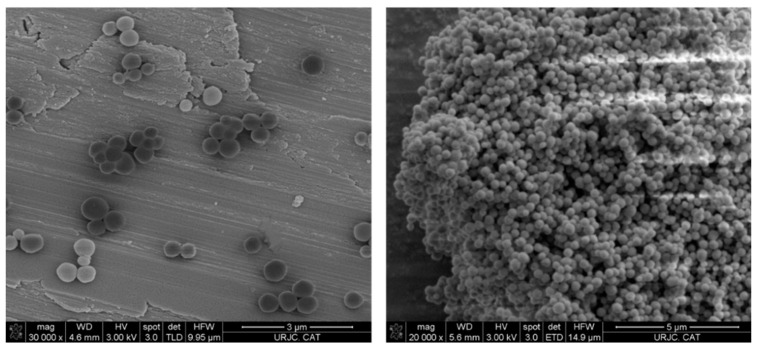
SEM images of starting MSN at different scales.

Transmission (TEM) and scanning microscopy analyses (SEM) show the morphology and size of the silica-based systems. In the TEM images, it can be seen how the particles have a hexagonal quasi-spherical shape (Figure 6). The histogram of the particle size of the materials exhibits high homogeneity with an average of 318 ± 32 nm. It is also observed that the particles show high dispersibility, not forming large aggregates, which is beneficial for their subsequent use in biological studies. Analysis via SEM shows a three-dimensional picture of the nanoparticles, indicating the homogeneous spherical morphology (Figure 7 and Figure S2).

### 3.2. Cell-Free Assays: Release Analysis and DNA Interaction Study

In order to confirm that the designed nanoparticulate systems act as “non-classical” drug delivery systems, a metal release study was carried out in a simulated physiological environment. All the materials show metal release below 0.6% with respect to their initial metal loading (Table 3), which indicates that the potential biological activity of these systems is not mainly due to the release of Pt(II) or Pd(II) soluble species in the target area, because the incorporation of the metal compounds via covalent binding makes the system very stable and not easily release the metal species. This can also be observed because after the incubation of the materials for long time periods (48 h), the concentration of Pt(II) or Pd(II) released in the medium does not significantly increase with respect to shorter incubation times. In fact, low metal release occurs after the first 3 h and after maintaining the incubation of the material in the physiological medium, a desorption–adsorption equilibrium of the released species occurs. This behavior is comparable with that found in previous studies of our research group, where metallodrug-functionalized silica-based materials with the metal compounds covalently bound did not show a release of metal species, even at high incubation times, acting as excellent cytotoxic agents [52].

**Figure 8:**
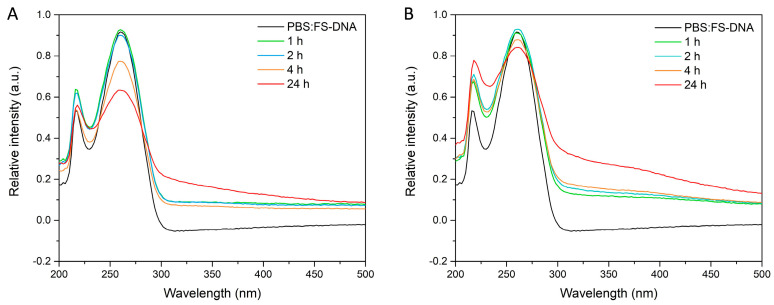
FS-DNA spectra at different incubation times with (**A**) MSN-PtTdTn and (**B**) MSN-PdTdTn.

The absorption spectra of the materials MSN-PdTdTn and MSN-PtTdTn in the presence of fish sperm DNA (FS-DNA) have been recorded at different incubation times. Figure 8 shows the UV spectra of each of the studied materials at different interaction time periods with free DNA. In the case of MSN-PtTdTn (Figure 8A), the maximum peaks of DNA absorption significantly decreased at the longest incubation time, maintaining the structure of the free DNA spectrum. This behavior is in agreement with that observed for other platinum complexes, which tend to form stable adducts across DNA guanines, leading cells to cell cycle arrest in the G2 phase [53]. However, the behavior of the Pd(II) complex after incubation with DNA is slightly different (Figure 8B). The highest intensity peak of DNA (around 260 nm) is slightly decreased in intensity at longer incubation times, but not as significantly as that for the Pt(II) complex. However, the peak at ca. 215 nm changes its intensity ratio with respect to the main peak as the incubation time increases, changing the morphology of the graph with respect to the free DNA. This behavior has previously been observed for other Pd(II) complexes, where a red shift occurs due to π → π*/n → π* transition bands, as the complex is incubated with more DNA [49]. This may indicate that the palladium complex functionalized in silica also interacts with DNA, which is one of the therapeutic targets, but whose mechanism of action is different from that of the platinum compound.

### 3.3. Biological Studies

**Figure 9:**
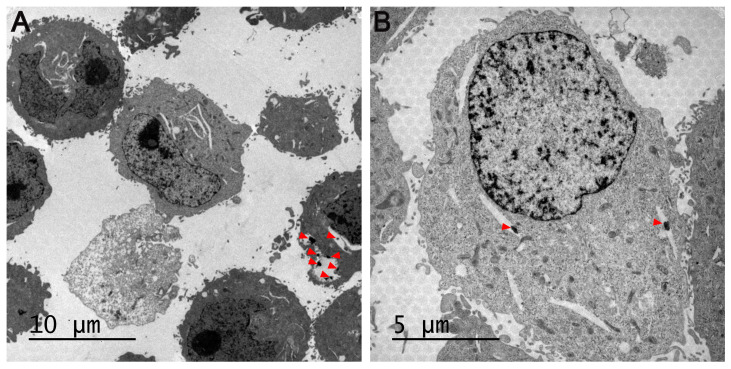
Cellular uptake of MSNs by HeLa. Cells were exposed to 10 µg/mL of palladium-containing MSNs for 24 h, and then the internalization of particles was imaged under TEM. Red arrowheads point to endocytosed MSNs. Scale bar: 10 µm (**A**) and 5 µm (**B**).

Regarding biological studies, the accumulation of MSNs inside cells was first confirmed through TEM, as judged by the presence of endocytosed nanoparticles (Figure 9) or clusters (Figure S3) of MSNs in the cytoplasm of HeLa cells. In this line, previous studies indicate that MSNs could enter cells via endocytosis and then release the metallodrugs to the cytosol [4,54]. Several endocytic mechanisms, such as clathrin-mediated endocytosis, caveolae-mediated endocytosis, and micropinocytosis, may impact the intracellular kinetics and the ultimate destiny of nanoparticles [55].

**Figure 10:**
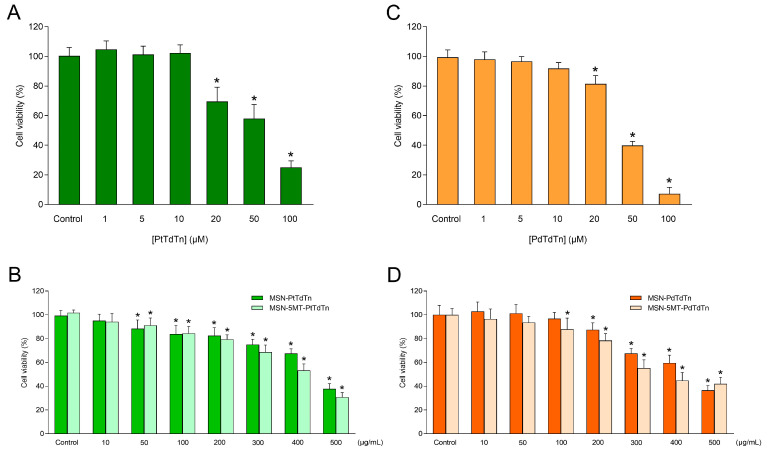
Dose–response curve of silica-based materials and the free compounds on cell viability. HeLa cells were stimulated with increasing concentrations of free PtTdTn (**A**), PdTdTn (**C**), platinum-containing MSNs (**B**), palladium-containing MSNs (**D**), or the vehicle (DMSO) for 24 h. Values are presented as means ± SD of 5 separate experiments and expressed as a percentage of control values. * *p* < 0.05 compared to its corresponding control value.

To verify the antitumor potential of the free compounds PtTdTn, PdTdTn and TdTn, their cytotoxic effect was tested at concentrations ranging from 1 to 100 µM in the cervical cancer cell line HeLa. The free PtTdTn complex and the ligand TdTn displayed similar cytotoxicity against HeLa cells (IC_50_ = 51.75 ± 2.43 and 51.59 ± 1.04 µM, respectively; Figure 10A and Figure S4A), while the PdTdTn complex possessed an enhanced cytotoxic effect (IC_50_ = 39.35 ± 0.86 µM; Figure 10C). Subsequently, the cytotoxicity of the silica-based materials against HeLa was also studied. The different materials were checked in the range of 10–500 µg/mL. MSN-PtTdTn and MSN-PdTdTn produced a significant cytotoxic effect starting from 50 µg/mL and 200 µg/mL, respectively (Figure 10B,D). In contrast, MSN-TdTn did not show any cytotoxic effect up to higher concentrations (>400 µg/mL; Figure S4B). Therefore, MSNs exhibited a dose-dependent cytotoxic effect against HeLa cells, the most cytotoxic material being MSN-PdTdTn with an IC_50_ value of 424.8 ± 9.59 µg/mL (Table 4). We computed the cytotoxic activity in terms of the whole material and as a function of the active metal loading of each material, so that both systems (materials and free compounds) could be compared in terms of the IC_50_ of their metal concentration (Table 4). Therefore, comparing the amount of metal required to obtain the same cytotoxic effect, the loading of the complexes into MSN-based delivery systems did not improve the biological activity of the free compounds. In fact, about three times less metal was required for the free PtTdTn complex (10.10 ± 0.79 vs. 31.44 ± 1.12 µg/mL; Table 4), and about two times less was required for the free PdTdTn (4.19 ± 0.18 vs. 8.92 ± 0.20 µg/mL; Table 4). However, in previous studies, MSN-based delivery systems incorporating platinum derivatives demonstrated improved cytotoxicity against HepG2 cells compared to free compounds [56]. The same was true for the MSN-TdTn used herein, which did enhance the cytotoxicity of the free ligand, requiring almost 70% less metal for MSN-TdTn (11.55 ± 0.77 vs. 17.12 ± 1.04 µg/mL; Table 4) to achieve the same effect.

On the other hand, the antitumor potential of the free 5MT was also evaluated, and melatonin was used as a reference compound. As shown in Figure S5, 5MT showed a relatively higher cytotoxic effect against HeLa than did melatonin (IC_50_ = 1.12 ± 0.02 vs. 1.26 ± 0.04 mM, respectively). After that, the biological activity of 5MT encapsulated in MSNs functionalized with the metallodrugs was also studied in HeLa cells (Figure 10 and Figure S4). The data obtained showed that MSNs conjugated with 5MT and their respective metallodrugs presented lower IC_50_ values than did MSNs loaded with the metallodrugs alone (Table 5). Comparing the amount of metal needed to obtain the same cytotoxic effect, the MSN-based delivery systems containing 5MT outperformed the biological activity of the free compounds, as ~3-fold less metal was needed for MSN-5MT-PtTdTn (3.41 ± 0.11 vs. 10.10 ± 0.79 µg/mL for PtTdTn), ~2-fold less was needed for MSN-5MT-PdTdTn (1.79 ± 0.04 vs. 4.19 ± 0.18 µg/mL for PdTdTn) and ~12-fold less was needed for MSN-5MT-TdTn (1.33 ± 0.07 vs. 17.12 ± 1.04 µg/mL for TdTn). The same trend was observed when comparing the amount of metal from metallodrug-loaded MSNs with metallodrug-containing MSNs conjugated to 5MT, as ~9-fold less metal was required for MSN-5MT-PtTdTn (3.41 ± 0.11 vs. 31.44 ± 1.12 µg/mL for MSN-PtTdTn), ~5-fold less was required for MSN-5MT-PdTdTn (1.79 ± 0.04 vs. 8.92 ± 0.20 µg/mL for MSN-PdTdTn) and ~9-fold less was required for MSN-5MT-TdTn (1.33 ± 0.07 vs. 11.55 ± 0.77 µg/mL for MSN-TdTn). Moreover, the administered amount of 5MT in MSN-5MT was 2.54 ± 0.40 µg/mL, i.e., ~84 times less than that of the free 5MT (212.8 ± 0.01 µg/mL; Table 5), thus indicating that the incorporation of small amounts of 5MT into the materials greatly improved the effectiveness of the complexes. These results agree with previous investigations combining different drugs in this kind of delivery systems. Thus, the combination of cisplatin together with doxorubicin [57] or nitric oxide [58] has been reported to enhance platinum cancer therapy in cell culture by reducing the dose of each drug.

**Figure 11:**
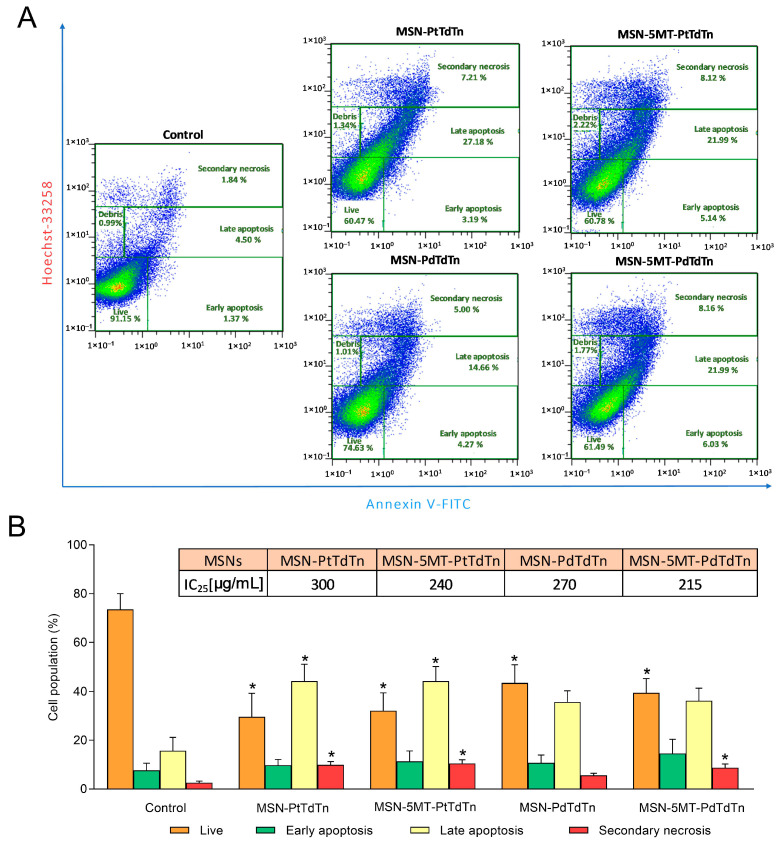
Platinum- and palladium-containing MSNs differentially affect the apoptosis of HeLa cells. Cells were treated with IC2_25_ of each MSN (as indicated) or the vehicle (control) for 24 h. (**A**) Representative cytograms of double-stained cells (Annexin V-FITC/Hoechst 33258) for each condition. (**B**) Histograms represent the percentage of different cell populations (means ± SD) of 4 separate experiments. * *p* < 0.05 compared to control values (Dunnett’s test).

Since 5MT-conjugated nanoparticles were the most effective delivery systems in terms of antitumor potential, the apoptosis-promoting effect of MSN-PtTdTn, MSN-5MT-PtTdTn, MSN-PdTdTn and MSN-5MT-PdTdTn was determined by analyzing phosphatidylserine externalization in double-stained (Annexin V-FITC/Hoechst 33258) HeLa cells. For this purpose, cells were challenged with the IC_25_ dose of the different MSNs (Figure 11) for 24 h. Platinum-containing materials induced a remarkable decrease in the proportion of live cells (26.14 ± 15.29% for MSN-PtTdTn and 32.12 ± 16.40% for MSN-5MT-PtTdTn; *p* < 0.05) at the expense of a significant increase in late apoptotic (44.26 ± 15.23% for MSN-PtTdTn and 44.24 ± 13.02% for MSN-5MT-PtTdTn; *p* < 0.05) and secondary necrotic (9.92 ± 2.94% for MSN-PtTdTn and 10.51 ± 3.22% for MSN-5MT-PtTdTn; *p* < 0.05) cell populations with respect to control cells (Figure 11). The palladium-containing materials followed a similar pattern, with a striking reduction in the proportion of live cells (43.45 ± 12.73% for MSN-PdTdTn and 39.41 ± 13.03% for MSN-5MT-PdTdTn; *p* < 0.05) at the expense of a notable rise in late apoptotic (35.71 ± 9.15% for MSN-PdTdTn and 36.15 ± 11.58% for MSN-5MT-PdTdTn) and secondary necrotic (8.71 ± 3.44% for MSN-5MT-PdTdTn; *p* < 0.05) cell populations with respect to control cells (Figure 11). These results are in concordance with preliminary findings, which indicated that the cytotoxic effect of PtTdTn and PdTdTn complexes was induced via ROS-mediated, caspase-dependent apoptosis [2]. Importantly, those materials conjugated with 5MT displayed better pro-apoptotic actions than their 5MT-free counterparts since 5MT-containing materials produced roughly the same proportion of apoptosis with a lower amount of metal (Figure 11B).

**Figure 12:**
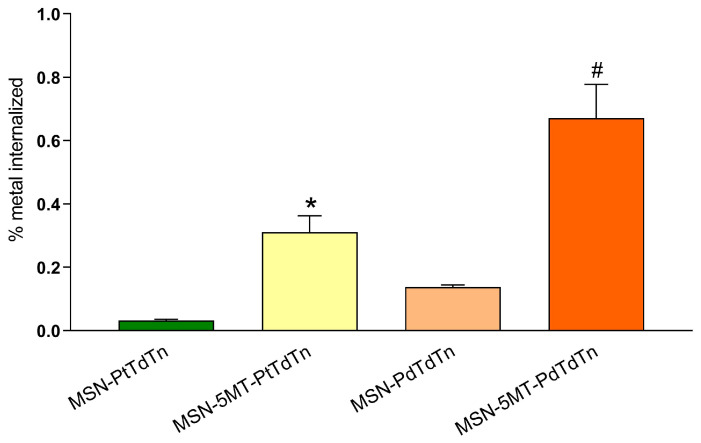
Platinum and palladium accumulation. HeLa cells were treated with 100 µg/mL of MSN-PdTdTn, MSN-5MT-PdTdTn, MSN-PtTdTn or MSN-5MT-PtTdTn for 3 h. Values represent means ± SD of 4 independent experiments and showed the percentage of metal internalized considering the amount of platinum or palladium loaded into each MSN system. * *p* < 0.05 compared to MSN-PtTdTn. # *p* < 0.05 compared to MSN-PdTdTn (Tukey’s test).

The enhanced cytotoxicity of MSNs functionalized with 5MT is hypothesized to be caused by the increased selectivity of the silica-based systems containing the metal complexes. Thus, we analyzed the accumulation of MSN-PtTdTn, MSN-5MT-PtTdTn, MSN-PdTdTn and MSN-5MT-PdTdTn in HeLa cells. In this sense, it becomes clear that the percentage of platinum internalized was much higher (up to 10-fold) when cells were challenged with MSN-5MT-PtTdTn (Figure 12). Palladium-containing nanoparticles followed the same trend but, in general, accumulated in higher amounts. In this case, the internalization of MSN-5MT-PdTdTn was up to five times higher than its counterpart lacking 5MT (Figure 12). Moreover, comparing the internalization of both metals, palladium was more efficiently internalized compared to platinum, which could explain the better cytotoxic effects observed for palladium-containing materials. Therefore, we suggest that the biological activity of MSNs is positively affected by functionalization with 5MT, which led to improved cellular uptake and accumulation and strengthened their potential therapeutic efficacy.

## 4. Conclusions

In this work, we report the use of MSNs to enhance the biological activity of two metal compounds based on a thiazoline ligand (PtTdTn and PdTdTn). The incorporation of these complexes covalently through protonolysis with the ligand 3-mercaptopropyltriethoxysilane was confirmed via various physicochemical techniques such as XRD, UV-vis in the solid state, BET and TG. The DNA interaction study of these materials showed that DNA is one of the main targets of the MSN-PtTdTn material, which is in agreement with the literature on the activity of platinum-based complexes. The MSN-PdTdTn material also reaches and affects DNA, but it might not be its main target. However, cell viability studies of these materials against the human epithelial cervix carcinoma cell line HeLa showed that, despite the biocompatibility of the starting material, the support of these complexes did not significantly improve their cytotoxic activity when comparing the activity of the metal loaded in the materials with the activity of the isolated complexes. Nevertheless, after the incorporation of a low amount of the melatonin derivative 5MT, the biological results were remarkable. The systems MSN-5MT-PtTdTn and MSN-5MT-PdTdTn show a higher accumulation of the materials in the cytoplasm of HeLa cells (up to 10 times in the case of the material with Pt(II) and 5 times in the case of Pd(II)), acting also as non-classical drug delivery systems, since metal release is less than 0.6%, so the activity of the designed systems is due to the whole material and not only to the active part of it. The systems conjugated with 5MT and their respective metallodrugs presented lower IC_50_ values than those found for MSNs loaded with the free metallodrugs (up to three times lower) and compared to the silica materials functionalized only with the metal complexes (up to nine times more active). The analysis of the apoptosis of the final materials showed an increase in late apoptosis and secondary necrosis in the 5MT-conjugated systems.

With all these, studies one can conclude that with the use of silica as a platform for the incorporation of metal complexes with anticancer activity and incorporating a molecule capable of increasing cellular accumulation at the same time (i.e., 5MT), the activity of the isolated complexes can be an interesting approach to improve the therapeutic activity of conventional treatments.

## Figures and Tables

**Table 1:** Quantification of the incorporation of 5MT, TdTn, Pt(II) and Pd(II).

Material	%5MT ^a^	%TdTn ^a^	%Pt ^b^	%Pd ^b^
MSN-TdTn	-	1.78	-	-
MSN-PtTdTn	-	11.58	6.80	-
MSN-PdTdTn	-	6.56	-	2.10
MSN-5MT	0.35	-	-	-
MSN-5MT-TdTn		0.21	-	-
MSN-5MT-PtTdTn		1.47	0.86	-
MSN-5MT-PdTdTn		1.52	-	0.49

^a^ quantified via TG; ^b^ quantified via ICP-AES.

**Table 2:** Textural properties of silica materials determined via BET analysis.

Material	S_BET_ (m^2^/g)	Pore Volume (cm^3^/g)	Pore Diameter (nm)
MSN	1064	0.80	2.8
MSN-5MT	901	0.54	2.8
MSN-TdTn	878	0.43	2.6
MSN-PtTdTn	856	0.41	2.8
MSN-PdTdTn	818	0.41	2.7
MSN-5MT-TdTn	761	0.47	2.9
MSN-5MT-PtTdTn	825	0.40	2.9
MSN-5MT-PdTdTn	808	0.39	2.8

**Table 3:** Mass percentage of palladium or platinum release at different incubation times.

Material	Time (h)	% Metal Release (±0.001)
MSN-PtTdTn	3	0.044
	24	0.044
	48	0.044
MSN-PdTdTn	3	0.024
	24	0.024
	48	0.024
MSN-5MT-PtTdTn	3	0.536
	24	0.360
	48	0.349
MSN-5MT-PdTdTn	3	0.102
	24	0.069
	48	0.126

**Table 4:** Cytotoxic effect after 24 h of treatment with MSN-PtTdTn, MSN-PdTdTn, MSN-TdTn and their free compounds expressed as a function of metal/ligand content. DMSO was used as a vehicle.

IC_50_ (±SD; µg/mL)			
Material/Compound	Total	Metal	Ligand
MSN	<1000	-	-
MSN-MP	<1000	-	-
MSN-PtTdTn	462.40 ± 16.52	31.44 ± 1.12	-
PtTdTn	30.96 ± 2.43	10.10 ± 0.79	-
MSN-PdTdTn	424.8 ± 9.59	8.92 ± 0.20	-
PdTdTn	20.05 ± 0.86	4.19 ± 0.18	-
MSN-TdTn	648.6 ± 43.26	-	11.55 ± 0.77
TdTn	17.12 ± 1.04	-	17.12 ± 1.04
Cisplatin	4.84 ± 1.01	3.14 ± 0.66	-

**Table 5:** Cytotoxic effect of MSNs functionalized with 5-methoxytryptamine (5MT) (MSN-5MT-PtTdTn, MSN-5MT-PdTdTn, MSN-5MT-TdTn, MSN-5MT) and their free compounds in HeLa cells.

IC_50_ (±SD; µg/mL)				
Material/Compound	Total	(Metal)	(Ligand)	(5MT)
MSN-MP-TEDTS	<1000	-	-	-
MSN-5MT-PtTdTn	394.90 ± 13.14	3.41 ± 0.11	-	-
MSN-5MT-PdTdTn	366.20 ± 8.69	1.79 ± 0.04	-	-
MSN-5MT-TdTn	632.9 ± 32.82	-	1.33 ± 0.07	-
MSN-5MT	724.7 ± 113	-	-	2.54 ± 0.40
5MT	1120 ± 0.02	-	-	212.80 ± 0.01

## Data Availability

The data that support the findings of this study are available from the corresponding author upon reasonable request.

## Supplementary Materials

The following supporting information can be downloaded at https://www.mdpi.com/article/10.3390/pharmaceutics16010092/s1. Figure S1. TG-DTG curves of MSN-TdTn (A), MSN-5MT (B) and MSN-5MT-TdTn (C). Figure S2. SEM micrographs of MSN material. Figure S3. Representative TEM images of clusters of palladium-containing MSNs (red arrowheads) internalized in HeLa cells. Figure S4. Dose-response curve of MSN-TdTn, MSN-5MT-TdTn and effects of the free ligand TdTn on cell viability. Figure S5. Kinetics of the dose–response curve of 5-methoxytriptamine (5MT) and melatonin.
