# Supplementary material for: Melatonin Derivative-Conjugated Formulations of Pd(II) and Pt(II) Thiazoline Complexes on Mesoporous Silica to Enhance Cytotoxicity and Apoptosis against HeLa Cells
Source: Pharmaceutics. 2024 Jan 10;16(1):92. doi: 10.3390/pharmaceutics16010092 (PMC10821514; doi:10.3390/pharmaceutics16010092)
Supplement: Supplementary file 1 [file pharmaceutics-16-00092-s001.zip › pharmaceutics-2744036-supplementary.pdf]

SUPPLEMENTARY MATERIAL

Melatonin Derivative-Conjugated Formulations of Pd(II) and Pt(II) Thiazoline Complexes on Mesoporous Silica to Enhance Cytotoxicity and Apoptosis against HeLa Cells

Samuel Estirado[1†], Diana Díaz-García[2†], Elena Fernández-Delgado[1], Emilio Viñuelas-Zahínos[3], Santiago Gómez-Ruiz[2]*, Sanjiv Prashar[2], Ana B. Rodríguez[1], Francisco Luna-Giles[3], José A. Pariente[1], Javier Espino[1]*

[1] Grupo de Investigación Neuroinmunofisiología y Crononutrición, Departamento de Fisiología, Facultad de Ciencias, Universidad de Extremadura, Avenida de Elvas s/n, 06006 Badajoz, Spain.

[2] COMET-NANO Group, Departamento de Biología y Geología, Física y Química Inorgánica, E.S.C.E.T., Universidad Rey Juan Carlos, Calle Tulipán s/n, 28933 Móstoles, Madrid, Spain.

[3] Grupo de Investigación Química de Coordinación, Departamento de Química Orgánica e Inorgánica, Facultad de Ciencias, Universidad de Extremadura, Avenida de Elvas s/n, 06006 Badajoz, Spain.

[†] These authors equally contributed.

*Correspondence to: jespino@unex.es; Tel. +34 924289796; santiago.gomez@urjc.es; Tel. +34 914888507

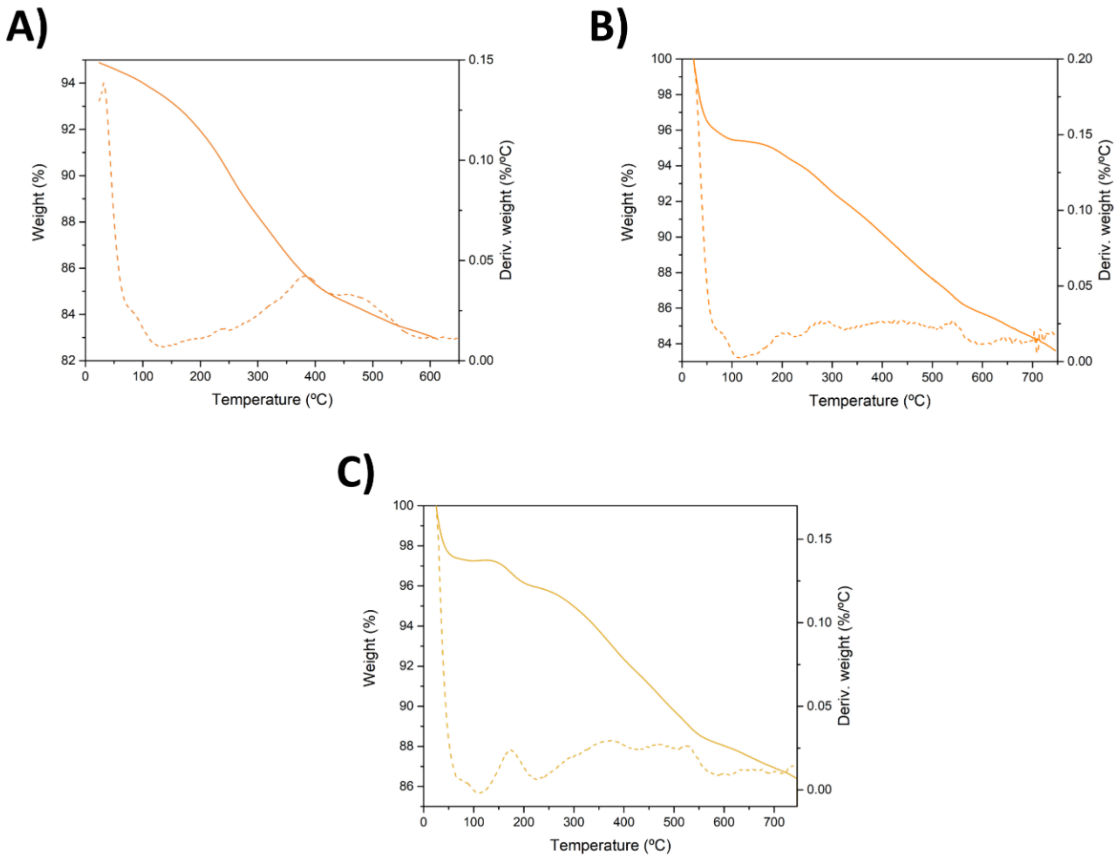

Figure S1. TG-DTG curves of MSN-TdTn (A), MSN-5MT (B) and MSN-5MT-TdTn (C).

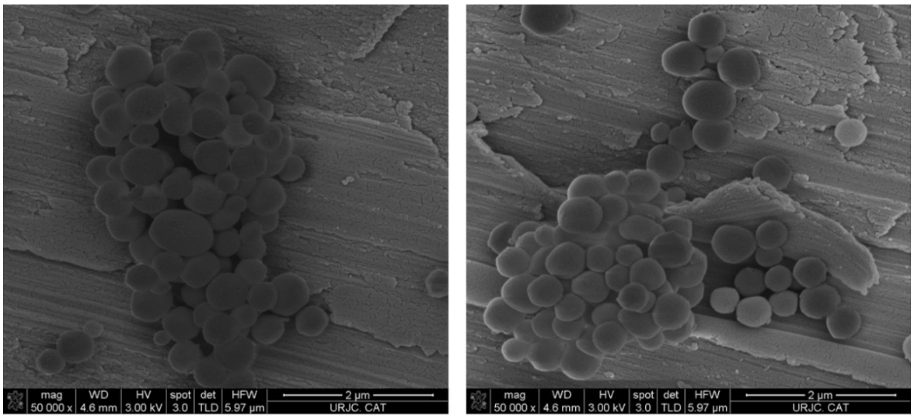

Figure S2. SEM micrographs of MSN material.

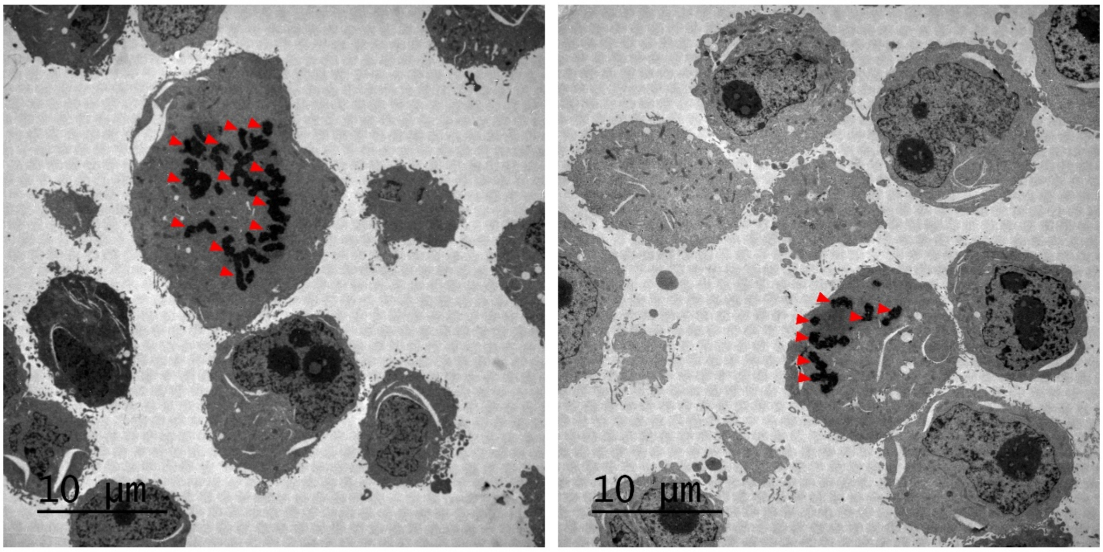

Figure S3. Representative TEM images of clusters of palladium-containing MSNs (red arrowheads) internalized in HeLa cells. Scale bar: 10 µm.

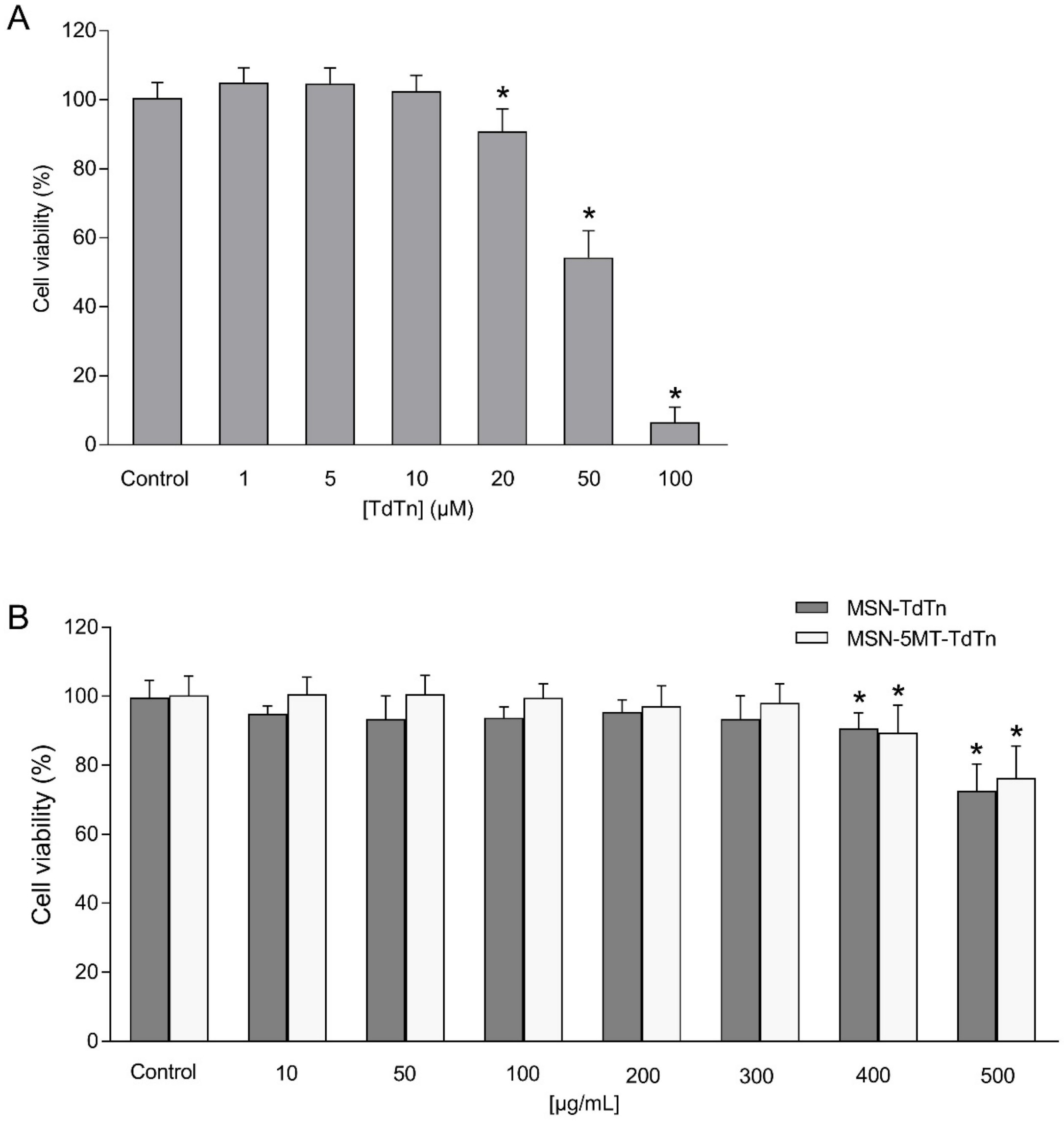

Figure S4. Dose-response curve of MSN-TdTn, MSN-5MT-TdTn and the free ligand TdTn on cell viability. A HeLa cells were stimulated with increasing concentrations of free TdTn (1-100 µM; **A**), silica-based materials (10-500 µg/mL; **B**) or the vehicle (DMSO) for 24 hours. Values are presented as means ± SD of 5 separate experiments and expressed as percentage of control values. *$P < 0.05$ compared to control values.

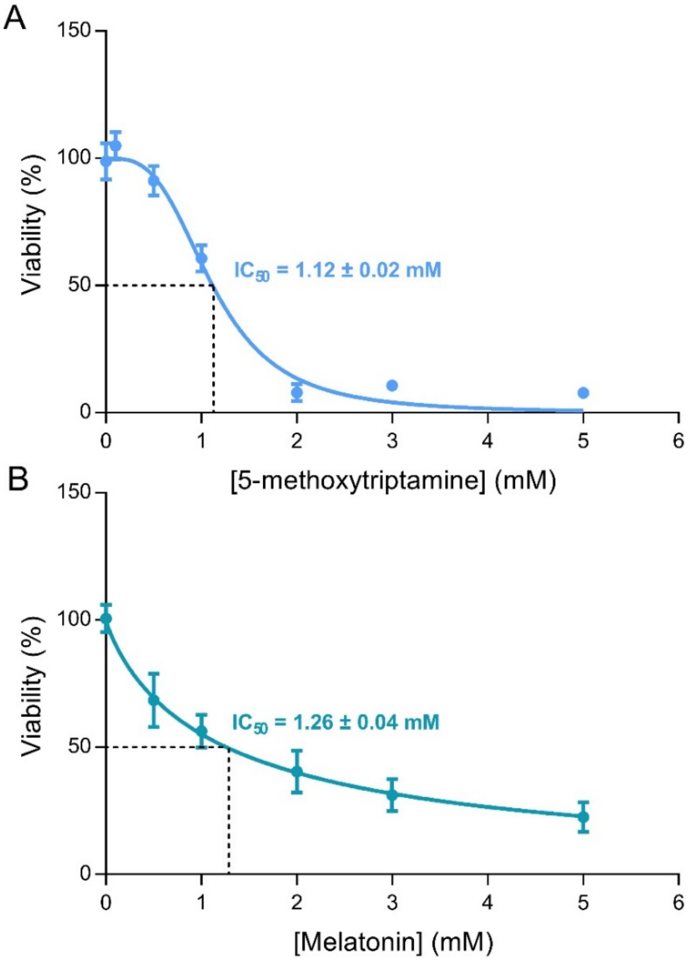

Figure S5. Kinetics of the dose-response curve of 5-methoxytriptamine (5MT) and melatonin. HeLa cells were stimulated with increasing concentrations (0.5, 1, 2, 3, and 5 mM) of 5MT (A) and melatonin (B), or the vehicle (DMSO) for 24 h**.** Values are presented as means ± SD of 5 separate experiments and expressed as percentage of control values. IC_{50} values were calculated by fitting the curve using a four-parameter, sigmoid dose-response equation.
